# Supplementary material for: Complexome profiling of the Chlamydomonas psb28 mutant reveals TEF5 as an early PSII assembly factor
Source: Plant Cell. 2025 Mar 17;37(6):koaf055. doi: 10.1093/plcell/koaf055 (PMC12164590; doi:10.1093/plcell/koaf055)
Supplement: koaf055_Supplementary_Data [file koaf055_supplementary_data.zip › psb28_interactive.zip!/plots/intensity/qualityPlots/peptides/SRP54L_Cre11.g479750.t1.2.html]

### Cre11.g479750.t1.2

Trivial name: SRP54L  
SeqCoverage [median]: 22.80  
Mass: 59.60 kDa  
  
 

| Sequence | MissedCleavage | Mass [Da] | wtIntensity | muIntensity | Score |
| --- | --- | --- | --- | --- | --- |
| AEAAIKEEDAQK | 1 | 1301.65 | 283.42 | 0.00 | 57.84 |
| ALLEADVSLPVVR | 0 | 1380.80 | 1264.97 | 0.00 | 107.79 |
| FVGVGEKMEALEPFYPER | 1 | 2097.02 | 1648.64 | 0.00 | 69.19 |
| GGAALSVR | 0 | 729.41 | 503.75 | 0.00 | 86.47 |
| ILGMGDVLTLYEK | 0 | 1450.77 | 255.01 | 0.00 | 77.06 |
| LGVDAVIIDTAGR | 0 | 1298.72 | 654.48 | 0.00 | 78.65 |
| LMAAYTSMK | 0 | 1014.49 | 373.44 | 0.00 | 48.79 |
| LQVDEGMMAELR | 0 | 1390.66 | 741.60 | 0.00 | 59.20 |
| VAQDSGKSEAEVTK | 1 | 1447.72 | 1572.88 | 0.00 | 70.41 |
| VLEGVTPDVQFIK | 0 | 1443.80 | 4527.32 | 0.00 | 118.28 |
|  |  |  | 11825.5 | 0.0 |  |
